# Computerised Methodologies for Non-Invasive Angiography-Derived Fractional Flow Reserve Assessment: A Critical Review

**DOI:** 10.1155/2020/6381637

**Published:** 2020-04-20

**Authors:** Anantharaman Ramasamy, Chongying Jin, Vincenzo Tufaro, Retesh Bajaj, Yakup Kilic, Hannah Safi, Rajiv Amersey, Daniel Jones, Ryo Torii, Alexandra Lansky, Anthony Mathur, Christos V. Bourantas, Andreas Baumbach

**Affiliations:** ^1^Department of Cardiology, Barts Heart Centre, Barts Health NHS Trust, London, UK; ^2^William Harvey Research Institute, Queen Mary University London, UK; ^3^Department of Mechanical Engineering, University College London, London, UK; ^4^Division of Cardiovascular Medicine, Department of Internal Medicine, Yale School of Medicine, New Haven, CT, USA; ^5^Institute of Cardiovascular Sciences, University College London, London, UK

## Abstract

Fractional flow reserve is the gold standard for assessing the haemodynamic significance of intermediate coronary artery stenoses. Cumulative evidence has shown that FFR-guided revascularisation reduces stent implantations and improves patient outcomes. However, despite the wealth of evidence and guideline recommendations, its use in clinical practice remains minimal. Patient and technical limitations of FFR as well as the need for intracoronary instrumentation, use of adenosine, and increased costs have limited FFR's applicability in clinical practice. Over the last decade, several angiography-derived FFR software packages have been developed which do not require intracoronary pressure assessment with a guidewire or need for administration of hyperaemic agents. At present, there are 3 commercially available software packages and several other non-commercial technologies that have been described in the literature. These technologies have been validated against invasive FFR showing good accuracy and correlation. However, the methodology behind these solutions is different—some algorithms are based on solving the governing equations of fluid dynamics such as the Navier–Stokes equation while others have opted for a more simplified mathematical formula approach. The aim of this review is to critically appraise the methodology behind all the known angiography-derived FFR technologies highlighting the key differences and limitations.

## 1. Introduction

Coronary angiography remains the established method for assessing the presence and severity of coronary artery disease. However, when an intermediate lesion (defined as diameter stenosis 40–90%) is identified on coronary angiography, further evaluation of its clinical significance is advised [1]. This is due to the discrepancy between anatomical narrowing and functional effect on the flow reserve in intermediate coronary stenosis [2, 3]. Andreas Gruentzig, the pioneer of coronary angioplasty, showed that post-angioplasty reduction in pressure across the stenotic lesion is a useful indicator of a successful procedure [4]. Today, fractional flow reserve (FFR) is regarded as the gold standard for evaluating the functional significance of intermediate lesions and guiding revascularisation [5]. There are robust data from multiple randomised controlled trials that support the use of FFR to guide revascularisation resulting in reduced major adverse cardiac events, improved patient outcomes, reduced stent implantation rates, and cost-effectiveness [6–8]. However, the use of FFR in clinical practice remains limited due to the invasive nature of the assessment, which has an increase in complication rates, and albeit small, often produces symptoms from the hyperaemic agents, as well as an increase in procedural time and associated costs.

One of the main limitations of two-dimensional (2D) coronary angiography is the presence of foreshortening and the difficulty in accurately assessing diffuse long and eccentric lesions. The introduction of 3D quantitative coronary angiography (3D-QCA), which combines two projections, addresses some of these limitations and has shown a stronger correlation with invasive functional assessment of coronary stenoses [9, 10]. More importantly, 3D-QCA also allows reconstruction of specific coronary geometries, which can then processed by computational methodologies that allow assessment of translesional pressure gradients.

Over the last decade, several angiography-derived methodologies that use 3D-QCA anatomic parameters to estimate FFR have been developed. These incorporate either blood flow simulation using computational fluid dynamic (CFD) techniques or a mathematical formula, which provides rapid calculation of the pressure drop across a lesion. The aim of this review is to critically appraise all the currently known angiography-derived FFR technologies in chronological order, focussing on their advantages and limitations.

## 2. Angiography-Derived FFR Software

### 2.1. Virtual Fractional Flow Reserve (vFFR)

The pioneering study of angiography-derived FFR was by Morris et al. [11] in 2011. The VIRTU-1 study included 35 lesions from 19 patients and examined the feasibility of computational workflow based on coronary angiography to predict FFR. The successful application of computational fluid dynamics techniques to computed tomographic coronary angiography (CTCA) images to calculate lesion-specific FFR and the superior resolution of coronary angiography encouraged the authors to focus on the application of CFD to create the first angiography-derived FFR software, the vFFR.

The methodology involves using 2D images from rotational coronary angiography to identify two projections from a similar phase of the cardiac cycle and that are at least 90° apart, which are reconstructed using the Phillips 3D workstation. The 3D reconstructed vessel is exported as a virtual reality modelling language file into a workflow. The inlet and outlets are defined and capped, and the surface is meshed into approximately 1 million tetrahedral elements. The proximal boundary condition is defined as the average proximal transient pressure waveform, and CFD analysis is performed using the commercially available software (ANSYS CFX). The solver is based on the Navier–Stokes continuity equation and principles of momentum conservation. The downstream boundary condition is developed by averaging the resistance and compliance values from all patients included in the study which is then applied as a generic condition according to the Windkessel model [12]. The simulation output is then used to derive vFFR results. The main limitations of vFFR include the use of rotational angiography which is not widely used in the clinical arena, the use of generic boundary conditions and the use of pulsatile CFD which translates into a very long computational times (>24 hours).

A few years later, the same group developed a novel “pseudotransient” analysis protocol for computing vFFR [13]. “Pseudotransient” refers to transient CFD results being approximated without the need to perform the time-consuming, fully transient analysis. The 3D reconstruction is similar to the previous vFFR simulation where the images are based on rotational coronary angiography. The CFD analysis is based on 2 steady-state analyses, which is used to derive linear and quadratic terms to characterise pressure and flow which are then used to derive vFFR. This new approach results in a significant improvement in the computation time from >24 hours to <4 minutes. The linear and quadratic equations used 9 parameters including coronary microvasculature resistance and compliance.

In 2019, the same group developed an extension to their vFFR software, the Virtual Coronary Intervention (VCI) [14]. The 3D reconstruction is based on 2 orthogonal views, as close to 90° from routine coronary angiography. End-diastolic frames are automatically chosen based on the electrocardiographic tracing. The 3D model is then exported to the VIRTUheart workflow software. The surface is meshed based on the previous vFFR algorithm. The inlet boundary is set as the mean aortic pressure taken during angiography, and a generic resistance value of 8.721*E* + 9 Pa/m^2^s^−1^ derived from the previous vFFR study is used to define the outlet boundary condition. Steady-state CFD computation is then performed using the commercially available (ANSYS CFX) software where the vFFR value can be calculated within a few minutes. The VCI allows virtual simulation of coronary stenting as part of the VIRTUheart software based on pre- and post-simulated vFFR calculation and prediction of physiological response to stenting.

### 2.2. Virtual Functional Assessment Index (vFAI)

**Figure 1:**
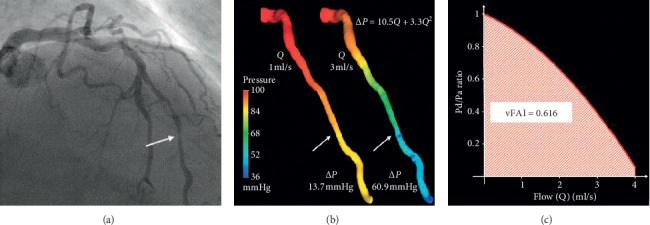
Functionally significant moderate lesion assessed by vFAI. (a) Coronary angiography image of a left anterior descending artery with an intermediate stenosis. The area with maximal diameter stenosis is marked with a white arrow. The haemodynamic significance of this lesion was assessed with invasive pressure wire showing FFR = 0.64 (haemodynamically significant). (b) The 3D-QCA reconstruction model and colour-coded pressure distribution map at two different flow rates, 1 ml/s and 3 ml/s. (c) Relationship between Pd/Pa ratio and coronary flow in the interrogated segment of the studied artery (reproduced with permission from Papafaklis et al. [15]).

After the introduction of vFFR, Papafaklis et al. [15] introduced a new angiography-derived FFR (Figure 1). The vFAI's methodology is based on CFD. The first stage of calculating vFAI is based on accurate 3D-QCA reconstruction using the validated CAAS 3D-QCA software (Pie Medical Imaging, Maastricht, the Netherlands). Pressure drop is linked to flow using linear and quadratic terms (Δ*P* = *f*_v_*Q* + *f*_s_*Q*^2^), where Δ*P* is the pressure gradient (mmHg), *Q* is the flow rate (ml/s), *f*_v_ is the coefficient of pressure loss due to viscous friction, and *f*_s_ is the coefficient of pressure loss due to flow separation. The 3D-QCA geometry is used to perform two separate CFD simulations to solve the *f*_v_ and *f*_s_ parameters. The arterial wall is considered rigid, and no-slip conditions are applied at the vessel wall with a reference pressure of 100 mmHg set at the inlet.

The vFAI does not require patient-specific blood flow measurements. The vFAI is calculated as the ratio of the area under curve for flow range between 0 and 4 mls/sec and is not identical to invasive FFR values.

The main limitations of vFAI are the exclusion of side branches and the assumption that coronary flow remains fixed across the length of the vessel. The average time required from extraction of 3D anatomy to completion of CFD is 15 minutes. The first prototype did not appear to be very user-friendly, and no further developments have been made since its introduction in 2014.

### 2.3. Quantitative Flow Reserve (QFR), Medis Medical Imaging System, Leiden, The Netherlands

**Figure 2:**
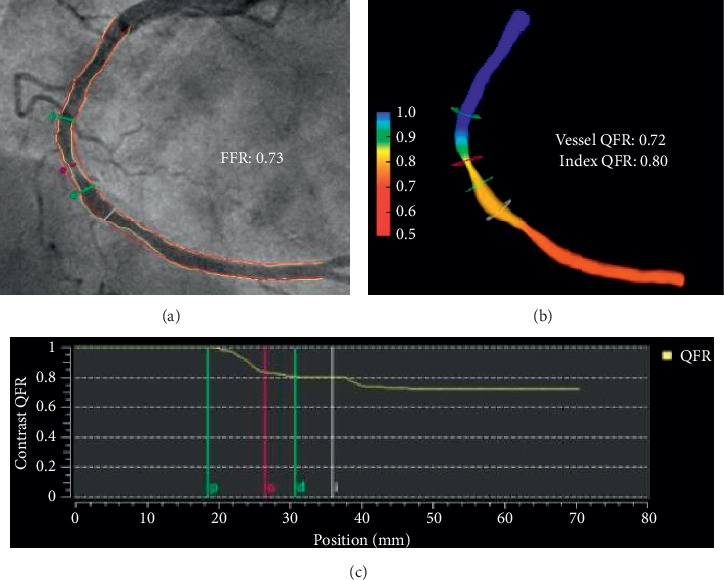
Haemodynamically significant intermediate stenosis assessed by QFR. (a) Coronary angiography showing a moderate lesion in the midright coronary artery with semiautomatic detection of lumen and vessel contours. The invasive FFR of this lesion showed haemodynamic significance (FFR = 0.73). (b) The computed colour-coded flow map of the reconstructed vessel. (c) Contrast-QFR pullback showing the pressure drop across the stenosis (p: proximal lesion marker; o: maximal lesion; d: distal lesion marker).

QFR is an angiography-derived translesional physiology assessment software that was introduced after VFR and vFAI (Figure 2). The initial study by Tu et al. in 2014 was based on a CFD approach [16]. This study (where the computed FFR is termed FFR_QCA_) included 77 vessels from 68 patients and showed good correlation (*r* = 0.81, *p* < 0.001) with invasive FFR. Furthermore, FFR_QCA_ showed a superior diagnostic accuracy to invasive FFR compared to minimum lumen area and diameter stenosis. Coronary angiography images were acquired at 15 or 30 frames/second; 3D-QCA was performed using the well-validated QAngioXA 3D software, and side branches with diameters larger than one-third of the main vessel were included in the reconstruction. When bifurcation lesions are present, the software reconstructs normal lumen borders, assuming there is no stenosis to determine the flow distribution between the main vessel and side branch. Multiple bifurcations in the same vessel are merged into a tree structure. The mean volumetric flow rate is calculated using the lumen volume of the reconstructed vessel divided by mean transport time, determined by thrombolysis in myocardial infarction (TIMI) frame count on hyperaemic acquisition obtained after adenosine infusion. The 3D geometry is meshed into tetrahedral cells, followed by the application of Navier–Stokes equations and non-linear partial differential equations (ANSYS Inc.). Blood is modelled as an incompressible and Newtonian fluid. Blood viscosity and density are derived from individual patient haematocrit information. Following the steady-state simulation, the FFR is calculated by dividing mean pressure at the outlet by mean pressure at the inlet.

Subsequent QFR studies have focussed on faster computation by adopting a mathematical approach [17]. Traditionally this was performed offline, although the workflow software can now be installed in the Cardiac Catheterisation Suite where angiographic images can be sent from the scanner to the workstation for fast analysis. The first step is similar to the earlier process, which involves creating a 3D reconstruction of the vessel using two angiographic projections, at least 25° apart with minimal foreshortening and good vessel opacification. The side branches are not reconstructed in this approach. The QFR software computation works on the following principles: (1) coronary pressure remains constant through normal coronary arteries; (2) a simple quadratic equation using coefficients derived from flow data is used to calculate the pressure drop across a stenosis based on the geometry and flow; (3) 3D-QCA is able to accurately characterise stenosis and vessel geometry; and (4) coronary flow velocity distal to the stenosis is similar to that proximal to the stenosis and the mass flow rate at each location along the vessel can be calculated using the mean flow velocity and 3D-QCA anatomic parameters. The QFR software provides 3 different computational values depending on the mean hyperaemic flow velocities used. The first is fixed-QFR (fQFR) where a fixed hyperaemic flow velocity of 0.35 m/s is used based on previous studies [16]. The second is contrast-QFR (cQFR) where frame count analysis on angiographic images without pharmacologically induced hyperaemia is used to calculate a flow velocity at non-hyperaemic conditions, which is then used to derive cQFR values. Finally, adenosine-QFR (aQFR) is similar to cQFR except that the angiographic projections used are acquired during hyperaemia (intravenous administration of adenosine) to provide the real hyperaemia flow velocity for aQFR computation. Both cQFR and aQFR use patient-specific flow derived from frame count, which proved to be superior to fQFR when validated [17]. Over the recent years, several QFR studies have been validated against invasive FFR showing good correlation and diagnostic accuracy [16–19]. QFR is the first commercially available software to be CE-marked and FDA-cleared.

### 2.4. Fractional Flow Reserve Derived from Coronary Angiography (FFRangio), CathWorks Ltd, Kfar-Saba, Israel

FFRangio provides functional angiography mapping of the coronary tree with superimposed colour-coded FFR values. FFRangio's computational method is based on rapid flow analysis [20]. The 3D coronary tree, including its centreline, cross-sections at each point along it path, and exact topology, is automatically created based on the geometry of at least 3 angiographic projections. The next step involves the user validation process where the 3D shape of the coronary arteries is projected back onto the 2D angiographic images for inspection. Following this, the software applies a compensation mechanism where it uses all the available projections to correct any *x*/*y*/*z* displacements due to breathing or patient movements during coronary angiography image acquisitions.

The coronary tree is then surfaced using a triangular mesh to display a 3D coronary model. The software automatically looks for a stenosis by performing systematic segment, branch, and junction level analysis. The coronary arterial network is modelled as an electrical circuit with each segment acting as a resistor. The vessel diameter and length determine its resistance. Each vessel's flow is based on its impact on overall resistance depending on the coronary tree arrangement, which is then lumped into a 3D model. Finally, haemodynamic evaluation of the stenosis is performed by considering the contribution of each narrowing to the total resistance and flow, which is then displayed as a colour-mapped coronary tree with FFR values at every stenosis. FFRangio is based on limited user interaction to reduce any discrepancy introduced by the user during processing.

The CathWorks FFRangio system is FDA-cleared and commercially available, with the unique advantage of providing reconstruction of the entire coronary tree with FFR values along each vessel.

### 2.5. Simplified Model of FFR Calculation (FFR_sim_)

FFR_sim_ is an angiography-derived FFR methodology that is based on 3D-QCA and classic fluid dynamics equations without using finite element analysis [21]. The coronary angiography images are used to create a 3D angiographic reconstruction offline using a validated, commercially available software (QAngio XA Research Edition, 1.0, Medis specials bv, Leiden, the Netherlands). FFR_sim_ calculation is based on the following simplified equation:

(1) $$\begin{array}{c} \Delta P=R*Q,\\ \Delta P_{\text{laminarflow}}=\left(\frac{8*\pi *n*L}{A^{2}}\right)*Q,\\ \Delta P_{\text{flowseperation}}=k_{\text{flowseperation}}*\frac{p}{0.266}*{\left(\frac{1}{\text{MLA}}-\frac{1}{\text{Adistal}}\right)}^{2}*Q^{2},\\ \text{where},\;k_{\text{flowseperation}}=1.21+0.08*\left(\frac{L_{\text{lesion}}}{2*\text{Dref}.\text{lesion}}\right), \end{array}$$

where Δ*P* = pressure gradient; *R* = resistance; *Q* = volumetric flow; *n* = blood viscosity; *L* = lesion length; and *p* = blood density.

Blood flow velocities are assumed identical proximal and distal to the stenosis at peak vasodilatation. The contrast flow along the vessel is calculated using the established TIMI frame count method. The flow rate in the main vessel will decrease proportionally to the cross-sectional area of the distal reference segment following a side branch. The distal mean arterial pressure (*P*_d_) is calculated by subtracting the pressure drop across the lesion (derived from the above equations) from the mean arterial pressure (*P*_a_). Finally, this allows calculation of FFR_sim_ which is defined as Pd/Pa across as stenosis. Tar et al. studied 68 vessels with single-vessel stenosis where FFR_sim_ shows strong correlation with invasive FFR (*r* = 0.86, *p* < 0.0001) [21]. The sensitivity and AUC of FFR_sim_ to detect haemodynamically significant lesions (FFR < 0.80) were 0.90 and 0.96, respectively.

### 2.6. Cardiovascular Angiographic Analysis Systems for Vessel Fractional Flow Reserve (CAAS-vFFR), Pie Medical Imaging, Maastricht, The Netherlands

**Figure 3:**
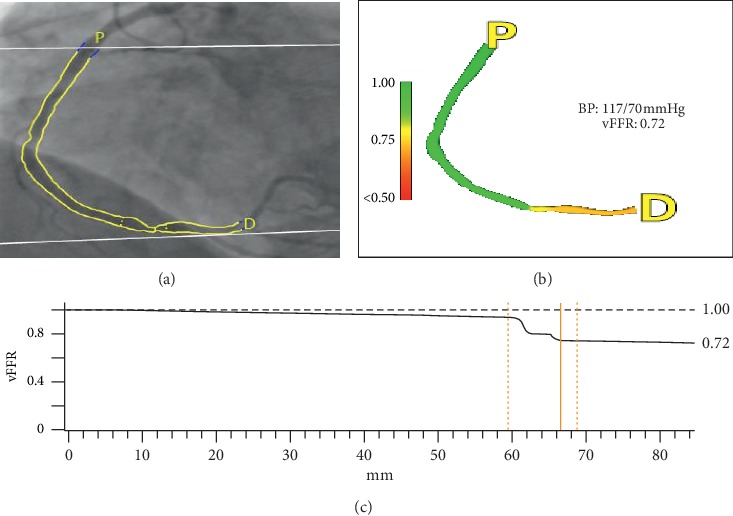
Haemodynamically significant intermediate stenosis assessed by CAAS-vFFR. (a) Coronary angiography demonstrating a moderate lesion in the distal right coronary artery with semiautomatic lumen border detection. The invasive FFR on this occasion is functionally significant (FFR = 0.74). (b) Colour-coded pressure drop map of the reconstructed 3D model. Aortic pressure acquired during coronary angiography (117/70 mmHg) is used for computation. (c) CAAS-vFFR pressure drop across the vessel highlighting the haemodynamic significance of the lesion.

The CAAS-vFFR is an angiography-derived FFR software (Figure 3) based on the reconstruction of the coronary artery anatomy from two orthogonal coronary angiography views (at least 30° difference in rotation/angulation) using the CAAS workstation [22]. ECG triggering, with an option for manual frame selection if needed, automatically performs temporal alignment of the cardiac cycle. Vessel contour detection is semiautomatic allowing for manual correction when necessary. The pressure drop calculation is instantaneous by assuming application of the physical law in coronary flow. The maximal hyperaemic blood flow in the proximal coronary artery is assumed to be preserved along the coronary of interest. The vFFR system calculates the pressure drop across a lesion based on the coronary flow behaviour physical laws, described by Gould [23] and Kirkeeide, but no further specific description of the methodology has been provided. Patient-specific aortic pressure is used during the analysis.

The FAST study showed that CAAS-vFFR had a diagnostic accuracy of 0.93 (*p* < 0.001) to detect lesions with FFR < 0.80. A good linear correlation between FFR and vFFR (*r* = 0.89, *p* < 0.001) was noted along with good reproducibility (interobserver variability *r* = 0.95, *p* < 0.001) [22]. The CAAS-vFFR is CE-marked in Europe, FDA-cleared, and commercially available for clinical use.

## 3. Discussion

Over the last decade, clinical studies have demonstrated the feasibility and reproducibility of angiography-derived FFR software. These new technologies appear to correlate well with invasive FFR showing excellent diagnostic accuracy when tested offline (Table 1). However, there are significant differences in methodology, assumptions, and automation between these technologies. The advantages and limitations of the angiography-derived FFR software are summarised in Table 2.

The angiography-derived FFR can be obtained from either blood flow simulation using CFD or by mathematical formula. A recent meta-analysis found no difference between the two approaches for the diagnostic accuracy of angiography-derived FFR [26]. The successful application of CFD to CTCA images to predict blood flow and lesion-specific FFR preceded the development of angiography-derived FFR software. This prompted the studies of Morris et al., Papafaklis et al., and Tu et al., the first 3 angiography-derived FFR studies to apply CFD to the reconstructed coronary anatomical model for FFR prediction. The ability of angiography-derived software to predict FFR is novel with higher accuracy than reported for CTCA-based methodology. This has to be at least partially attributed to the superior resolution of invasive coronary angiography compared to computed tomography. The CFD approach is based on solving the fundamental governing equations of fluid dynamics—the continuity, momentum, and energy equations. The complex CFD modelling process requires prolonged computation time which has been a challenge to overcome for clinical applications. The first VIRTU-1 study by Morris et al. using transient (pulsatile) flow conditions required 24 hours of computing time for each vessel analysed. Subsequently, Tu et al. applied a steady-state flow simulation by assuming that the average pressure distribution over a cardiac cycle is no difference between these two approaches. This reduced computational time to 5 minutes per vessel representing a breakthrough in the field.

The complexity and time-consuming process of CFD-based methodology allowed the development of mathematical formula-based methodology. This allowed easier and faster calculation of pressure drop across a coronary stenosis. Two years following their initial study, Tu et al. developed a new methodology that avoided solving the traditional Navier–Stokes equation. QFR calculates the pressure drop across a stenosis using a simple quadratic equation using coefficients derived from flow data from their previous experimental models [17]. The instantaneous FFR computation meant that mathematical-based technologies became popular although subsequent studies applied a variety of different mathematical formulas. The CAAS-vFFR software calculates pressure drop across a stenosis by applying physical laws of coronary flow by Lance Gould and Kirkeeide. Both studies have reported good correlation and diagnostic accuracy compared to invasive FFR although there has no comparison between the different formula applications.

Segmentation is a key process of accurate 3D reconstruction. Most of the angiography-derived FFR software packages require just 2 projections acquired from routine coronary angiography. Biplane angiography is ideal but is not readily available in most catheterisation laboratories. However, most software try to correct for patient movement between angiography image acquisitions. The vFFR software is unique where it requires rotational coronary angiography, which offers multiple views to select the ideal projections for 3D reconstruction. However, rotational angiography is not widely used in clinical practice.

The inclusion of side branches as part of the 3D reconstruction has benefits and limitations. FFRangio provides a 3D reconstruction of the entire coronary tree, including side branches, with FFR values along each vessel. This is an attractive feature as it helps cardiologists plan individualised treatments, especially for patients with multivessel disease. The initial study by Tu et al. in 2014 included side branches as part of the 3D reconstruction. However, subsequently, the same group evolved the QFR software to focus on reconstruction of the main vessel only to significantly reduce the complexity and computation time. The authors concluded that the tapering reference diameter from 3D-QCA data can predict the decreasing mass flow rate along the vessel as the side branches are taking off while the mean flow velocity remains constant.

Most, if not all of the above-described methodologies are based on significant assumptions. Poiseuille law assumes a laminar flow travelling through a circular tube with constant cross-sectional area where the fluid is incompressible and Newtonian. Blood is non-Newtonian and the cross-sectional area of coronary arteries changes, even in a healthy vessel from proximal to distal end of the vessel. Despite these assumptions, the FFRangio showed good diagnostic accuracy when compared with invasive FFR. It is worth noting that FFRangio calculates the maximal flow rate in the stenosis compared to maximal flow rate in the absence of stenosis where else invasive FFR and several other angiography-derived models have focussed on the pressure gradients across a stenosis.

The coronary microcirculation, resistance, and functional capacity of the distal myocardial bed have a significant influence on coronary flow during hyperaemia [27]. vFAI is based on the fixed universal hyperaemic flow limit of 4 mls/sec which is an oversimplification of complex boundary conditions. Myocardial damage following myocardial infarction, presence of collateral vessels, and raised myocardial resistance due to chronic conditions such as diabetes mellitus and hypertension can make vFAI assessments tricky and potentially inaccurate. A recent study of 300 patients showed that, in patients with coronary microvascular dysfunction, defined as high index of microcirculatory resistance (IMR) of >23 units, the positive predictive value of QFR significantly decreases from 93% to 67% [28]. This is likely due to microcirculatory involvement which is known to carry a higher risk of adverse outcomes [29]. Furthermore, QFR calculates pressure gradients by measuring flow whereas FFR estimates flow by measuring pressure gradients. Despite this, the negative predictive value of the patients with high IMR in this study remained high at 87% highlighting the value of QFR in identifying low-risk patients.

Similarly, because vFFR assumes standardized downstream resistance, vFFR values are likely to underestimate the significance of a coronary stenosis in cases where the microcirculatory resistance in the distal coronary bed is elevated. The fQFR model of QFR relies on a hyperaemic fixed velocity of 0.35 m/s, irrespective of the coronary vessels. This is in contrast to some reports where the left anterior descending artery flow was noted to be significantly higher (0.62 m/s) than other vessels [30]. Given the flow and pressure relationship of the quadratic equation, it is not surprising that the cQFR and aQFR both showed better correlation to invasive FFR when compared to fQFR. Additionally, there may be a difference between coronary flow and perfusion between the left and right coronary arteries due to the systolic pressure differences between the ventricles [22]. This may be relevant for QFR analysis, specifically cQFR and aQFR where the frame counting is key during the computation of QFR values. However, no information is available detailing this potential pitfall in the published QFR literature.

## 4. Conclusion

Several angiography-derived FFR methodologies have shown good correlation and accuracy with invasive FFR to detect haemodynamically significant lesions, independent of CFD-based or simplified mathematical approaches. All methods have some variation in their assumptions for computing FFR values. At present, 3 angiography-derived FFR software packages (QFR, CAAS-vFFR, and FFRangio) are commercially available, based on offline validation studies that demonstrate fast and reliable 3D reconstruction and FFR computation. Clinical outcomes studies are currently underway (FAVOR III EJ: NCT03729739 and FAVOR III China: NCT03656848) and highly anticipated to incorporate angiography-derived FFR technologies in our daily clinical decision-making and guidelines. This will revolutionise our interpretation of diagnostic angiography by improving patient risk stratification through greater penetration in clinical practice and ultimately improve patient outcomes.

## Figures and Tables

**Table 1:** Landmark studies investigating the accuracy of angiography-derived FFR software against invasive FFR.

Study	Patients (lesions)	Diabetes mellitus (%)	Previous myocardial infarction	Lesion length (mm)	Diameter stenosis (%)	Prevalence of ischaemia (FFR≤0.80)	Mean/median FFR	Correlation	AUC	Exclusion criteria
*VFR*										
Morris et al. [11]	19 (19)	5	5%	NA	NA	NA	NA	0.84	0.97	Previous MI
										Significant LMS stenosis
										Previous CABG or PCI
										Too obese for rotational coronary angiography
Morris et al. [13]	20 (73)	30	45%	NA	NA	NA	NA	0.77	1	Previous CABG
										Chronic total occlusion
										Acute presentation within the last 60 days
Gosling et al. [14]	54 (59)	22	43%	NA	NA	NA	0.66 ± 0.14	0.87	0.93	Previous CABG
										Chronic total occlusion
										Acute presentation within the last 60 days

*vFAI*										
Papafaklis et al. [15]	120 (139)	28	31%	59.4 ± 21.0	61.4 ± 13.1	37%	0.84 (IQR 0.75–0.90)	0.78	0.92 (0.86–0.96)	Significant LMS disease
										Bifurcation lesions
										Infarct-related vessels
										Vessels with ostial stenosis
										Previous CABG

*QFR*										
Tu et al. [16]^*∗*^	77 (68)	29	22%	NA	46.6 ± 7.3	30%	0.82 ± 0.10	0.81	0.93 (0.86–0.99)	Interrogated vessel with significant overlap or foreshortening (>90%)
										Hyperaemic image quality insufficient for frame counting
										Mean pressure in the guiding catheter or blood haematocrit are unavailable
Tu et al. [17]	73 (84)	27	32%	NA	46.1 ± 8.9	32%	0.84 ± 0.08	0.77	0.92 (0.85–0.97)	Ostial LMS or RCA lesion
										Prior CABG
Xu et al. [19]	304 (328)	28	16%	13.1 ± 6.4	46.5 ± 11.3	34%	0.82 ± 0.12	0.86	0.96 (0.94–0.98)	Ostial lesions <3 mm from aorta
										Severe vessel overlap or tortuosity
										Luminal reduction due to myocardial bridge
										Poor angiographic image quality
										Main vessels with stenotic side branches downstream of interrogated lesion
Westra et al. [24]	172 (255)	10	NA	NA	50 ± 12	36%	0.82 ± 0.11	0.70	0.86 (0.81–0.91)	Ostial LMS or RCA stenosis
										<2 projections of visible stenosis
										No nitroglycerin administration
										Pressure wire position not documented
Westra et al. [18]	272 (317)	29	NA	9.64 (IQR 7.53–13.76)	45 ± 10	33%	0.83 ± 0.09	0.83	0.92 (0.89–0.96)	Medina type 1,1,1 and 1,0,1 lesions
										Aorto-ostial lesions
										Poor angiographic quality
										No nitroglycerin administration
										Stenosis at or near large diameter shifts (>1 mm)
										Severe tortuosity or overlap

*FFRangio*										
Pellicano et al. [20]	184 (203)	32	19%	NA	NA	NA	0.81 ± 0.11	0.81	0.93	Ostial LMS or RCA stenosis
										LMS stenosis
										In-stent restenosis of the target vessel
										Previous CABG
										Diffuse coronary artery disease
Fearon et al. [25]	301 (319)	32	NA	NA	51 ± 10	43%	0.81 ± 0.13	0.80	0.94 (0.92–0.97)	LMS stenosis >50%
										Recent (<12 months) stent placement in the target vessel
										In-stent restenosis
										Severe diffuse disease
										Target vessel supplied by collaterals
										Inadequate angiographic image quality

*FFRsim*										
Tar et al. [21]	64 (68)	27	52%	NA	46	NA	NA	0.86	0.96 (0.91–1)	Previous CABG
										Bifurcation lesions
										Ostial LMS stenosis

*vFFR*										
Masdjedi et al. [22]	100 (100)	26	NA	20 ± 13	37 ± 13	42%	0.82 ± 0.08	0.89	0.93 (0.88–0.97)	LMS stenosis
										Previous CABG with collaterals
										Cardiogenic shock or severe haemodynamic instability
										STEMI or lesions containing thrombus

AUC, area under the curve; CABG, coronary artery bypass grafting; FFR, fractional flow reserve; LMS, left main stem; MI, myocardial infarction; PCI, percutaneous coronary intervention; PCI, percutaneous coronary intervention; RCA, right coronary artery. *Note*. Tu (2014)^*∗*^—FFR_QCA_ study.

**Table 2:** Key advantages and limitations of all the angiography-derived FFR software.

Angiography-derived FFR methodologies	Advantages	Limitations
vFFR	Fast FFR computation^*∗*^	Need for rotational coronary angiography
	Incorporation of coronary microvascular information	Generic boundary condition

vFAI	First methodology with a reasonable computation time with a clinical potential	Single-vessel analysis
		Assumption of static coronary flow across the vessel

QFR	Instantaneous FFR computation	Single vessel analysis
	User-friendly interface	Unsuitable for aorto-ostial lesion assessment
	Estimates flow from patient-specific data and TIMI frame count	Need for nitroglycerin administration prior to angiography image acquisition
	Extensively validated against FFR	

FFRangio	Complete functional assessment of coronary tree	Unsuitable for aorto-ostial lesion assessment
	Fast FFR computation	
	Able to reconstruct coronary artery anatomy using more than 2 angiographic projections	

FFRsim	Simplified equation for FFR calculation	Single validation study
	Flow distribution before and after bifurcation was calculated	TIMI frame count used following administration of intracoronary adenosine

CAAS-vFFR	Instantaneous FFR computation	Single-vessel analysis
	User-friendly interface	Unsuitable for aorto-ostial lesion assessment
		Need for invasive blood pressure information

^*∗*^Fast FFR computation is based on the latest VIRTU-Fast study [13].

## References

[1] Neumann F. J., Sousa-Uva M., Ahlsson A. (2019). 2018 ESC/EACTS guidelines on myocardial revascularization. *European Heart Journal*.

[2] Park S.-J., Kang S.-J., Ahn J.-M. (2012). Visual-functional mismatch between coronary angiography and fractional flow reserve. *JACC: Cardiovascular Interventions*.

[3] Safi M., Eslami V., Namazi M. H. (2016). Visual-functional mismatch between coronary angiography, fractional flow reserve, and quantitative coronary angiography. *The International Journal of Angiology*.

[4] Anderson H. V., Roubin G. S., Leimgruber P. P. (1986). Measurement of transstenotic pressure gradient during percutaneous transluminal coronary angioplasty. *Circulation*.

[5] Pijls N. H. J., De Bruyne B., Peels K. (1996). Measurement of fractional flow reserve to assess the functional severity of coronary-artery stenoses. *New England Journal of Medicine*.

[6] Bech G. J. W., De Bruyne B., Pijls N. H. J. (2001). Fractional flow reserve to determine the appropriateness of angioplasty in moderate coronary stenosis. *Circulation*.

[7] Tonino P. A. L., De Bruyne B., Pijls N. H. J. (2009). Fractional flow reserve versus angiography for guiding percutaneous coronary intervention. *New England Journal of Medicine*.

[8] De Bruyne B., Pijls N. H. J., Kalesan B. (2012). Fractional flow reserve-guided PCI versus medical therapy in stable coronary disease. *New England Journal of Medicine*.

[9] Yong A. S. C., Ng A. C. C., Brieger D., Lowe H. C., Ng M. K. C., Kritharides L. (2011). Three-dimensional and two-dimensional quantitative coronary angiography, and their prediction of reduced fractional flow reserve. *European Heart Journal*.

[10] Bourantas C. V., Tweddel A. C., Papafaklis M. I. (2009). Comparison of quantitative coronary angiography with intracoronary ultrasound. Can quantitative coronary angiography accurately estimate the severity of a luminal stenosis?. *Angiology*.

[11] Morris P. D., Ryan D., Morton A. C. (2013). Virtual fractional flow reserve from coronary angiography: modeling the significance of coronary lesions. *JACC: Cardiovascular Interventions*.

[12] Westerhof N., Lankhaar J.-W., Westerhof B. E. (2009). The arterial Windkessel. *Medical & Biological Engineering & Computing*.

[13] Morris P. D., Silva Soto D. A., Feher J. F. A. (2017). Fast virtual fractional flow reserve based upon steady-state computational fluid dynamics analysis. *JACC: Basic to Translational Science*.

[14] Gosling R. C., Morris P. D., Silva Soto D. A., Lawford P. V., Hose D. R., Gunn J. P. (2019). Virtual coronary intervention. *JACC: Cardiovascular Imaging*.

[15] Papafaklis M. I., Muramatsu T., Ishibashi Y. (2014). Fast virtual functional assessment of intermediate coronary lesions using routine angiographic data and blood flow simulation in humans: comparison with pressure wire - fractional flow reserve. *EuroIntervention*.

[16] Tu S., Barbato E., Köszegi Z. (2014). Fractional flow reserve calculation from 3-dimensional quantitative coronary angiography and TIMI frame count. *JACC: Cardiovascular Interventions*.

[17] Tu S., Westra J., Yang J. (2016). Diagnostic accuracy of fast computational approaches to derive fractional flow reserve from diagnostic coronary angiography. *JACC: Cardiovascular Interventions*.

[18] Westra J., Andersen B. K., Campo G. (2018). Diagnostic performance of in-procedure angiography-derived quantitative flow reserve compared to pressure-derived fractional flow reserve: the FAVOR II Europe-Japan study. *Journal of the American Heart Association*.

[19] Xu B., Tu S., Qiao S. (2017). Diagnostic accuracy of angiography-based quantitative flow ratio measurements for online assessment of coronary stenosis. *Journal of the American College of Cardiology*.

[20] Pellicano M., Lavi I., De Bruyne B. (2017). Validation study of image-based fractional flow reserve during coronary angiography. *Circulation Cardiovascular Interventions*.

[21] Tar B., Jenei C., Dezsi C. A. (2018). Less invasive fractional flow reserve measurement from 3-dimensional quantitative coronary angiography and classic fluid dynamic equations. *EuroIntervention*.

[22] Masdjedi K., van Zandvoort L. J. C., Balbi M. M. (2019). Validation of 3-dimensional quantitative coronary angiography based software to calculate fractional flow reserve: fast assessment of STenosis severity (FAST)-study. *EuroIntervention*.

[23] Gould K. L., Kelley K. O., Bolson E. L. (1982). Experimental validation of quantitative coronary arteriography for determining pressure-flow characteristics of coronary stenosis. *Circulation*.

[24] Westra J., Tu S., Winther S. (2018). Evaluation of coronary artery stenosis by quantitative flow ratio during invasive coronary angiography: the WIFI II study (Wire-Free functional imaging II). *Circulation Cardiovascular Imaging*.

[25] Fearon W. F., Achenbach S., Engstrom T. (2019). Accuracy of fractional flow reserve derived from coronary angiography. *Circulation*.

[26] Collet C., Onuma Y., Sonck J. (2018). Diagnostic performance of angiography-derived fractional flow reserve: a systematic review and Bayesian meta-analysis. *European Heart Journal*.

[27] Kern M. J., Lerman A., Bech J.-W. (2006). Physiological assessment of coronary artery disease in the cardiac catheterization laboratory. *Circulation*.

[28] Mejía-Rentería H., Lee J. M., Lauri F. (2018). Influence of microcirculatory dysfunction on angiography-based functional assessment of coronary stenoses. *JACC: Cardiovascular Interventions*.

[29] van de Hoef T. P., van Lavieren M. A., Damman P. (2014). Physiological basis and long-term clinical outcome of discordance between fractional flow reserve and coronary flow velocity reserve in coronary stenoses of intermediate severity. *Circulation: Cardiovascular Interventions*.

[30] Ofili E. O., Kern M. J., St Vrain J. A. (1995). Differential characterization of blood flow, velocity, and vascular resistance between proximal and distal normal epicardial human coronary arteries: analysis by intracoronary Doppler spectral flow velocity. *American Heart Journal*.

